# α-Thalassemia Associated with Hb Instability: A Tale of Two Features. The Case of Hb Rogliano or α1 Cod 108(G15)Thr→Asn and Hb Policoro or α2 Cod 124(H7)Ser→Pro.

**DOI:** 10.1371/journal.pone.0115738

**Published:** 2015-03-02

**Authors:** Maria Grazia Bisconte, Mercedes Caldora, Gennaro Musollino, Giovanna Cardiero, Angela Flagiello, Gaetana La Porta, Laura Lagona, Romeo Prezioso, Gabriele Qualtieri, Carlo Gaudiano, Emilia Medulla, Antonello Merlino, Piero Pucci, Giuseppina Lacerra

**Affiliations:** 1 U.O.S. Microcitemia e patologia del globulo rosso, O.O.C. Ematologia A.O. Cosenza, Italy; 2 Laboratorio Specialistico di Ematologia, P.O. San Giovanni Bosco A.S.L. NA1, Napoli, Italy; 3 Istituto di Genetica e Biofisica “Adriano Buzzati-Traverso”- Consiglio Nazionale delle Ricerche (CNR), Napoli, Italy; 4 Dipartimento di Scienze Chimiche and Ceinge Biotecnologie Avanzate, Università degli Studi “Federico II”, Napoli, Italy; 5 U.O.D. di Thalassemia, ARNAS “Garibaldi”, Catania, Italy; 6 Ospedale Civile, Centro per la lotta contro le Microcitemie, Matera, Italy; 7 Dipartimento di Scienze Chimiche, Università degli Studi “Federico II” and Istituto di Biostrutture e Bioimmagini, CNR Napoli, Italy; University of South Florida College of Medicine, UNITED STATES

## Abstract

We identified two new variants in the third exon of the α-globin gene in families from southern Italy: the Hb Rogliano, α1 cod108 ACC>AAC or α1[α108(G15)Thr→Asn] and the Hb Policoro, α2 cod124 TCC>CCC or α2[α124(H7)Ser→Pro]. The carriers showed mild α-thalassemia phenotype and abnormal hemoglobin stability features. These mutations occurred in the G and H helices of the α-globin both involved in the specific recognition of AHSP and β1 chain. Molecular characterization of mRNA, globin chain analyses and molecular modelling studies were carried out to highlight the mechanisms causing the α-thalassemia phenotype. The results demonstrated that the α-thalassemia defect associated with the two Hb variants originated by different defects. Hb Rogliano showed an intrinsic instability of the tetramer due to anomalous intra- and inter-chain interactions suggesting that the variant chain is normally synthesized and complexed with AHSP but rapidly degraded because it is unable to form the α1β1 dimers. On the contrary in the case of Hb Policoro two different molecular mechanisms were shown: the reduction of the variant mRNA level by an unclear mechanism and the protein instability due to impairment of AHSP interaction. These data highlighted that multiple approaches, including mRNA quantification, are needed to properly identify the mechanisms leading to the α-thalassemia defect. Elucidation of the specific mechanism leads to the definition of a given phenotype providing important guidance for the diagnosis of unstable variants.

## Introduction

Hemoglobin A (HbA), the adult oxygen carrier, is composed of 2α and 2β-globin chains. Mutations in the globin genes cause blood disorder by affecting the production, stability or functional properties of the Hb tetramers. Mutations that inhibit protein folding, interactions with heme or with subunit assembly (termed α1β1 or α1β2), render the proteins susceptible to denaturation and proteolytic degradation [1,2]. Some Hbs are designated as hyperunstable because they are destroyed so rapidly that they are barely detectable or totally undetectable in the hemolysate. These Hbs usually result from mutations localized in the third exon, in regions coding for the α1β1 contact. Some of these hyperunstable variants may be detected after splenectomy in the hemolysate [2].

The identification of the α-hemoglobin stabilizing protein (AHSP) has provided new insights into the human anemias associated with HbA instability [3,4]. In the last few years increasing attention has been focused on understanding the effect of the α-globin mutation on the interaction with the α-AHSP chaperone, showing that in some mutants the α-thalassemia defect is caused by an impaired binding of AHSP to the α-globin chain variants [5–7].

Although it is generally agreed that clinical effects are related to an abnormal protein, it is conceivable that in same cases the mutation in the globin gene may also impair expression mechanisms producing aberrant mRNA that could be either inadequately processed or be unstable and degraded [8,9].

**Fig 1:**
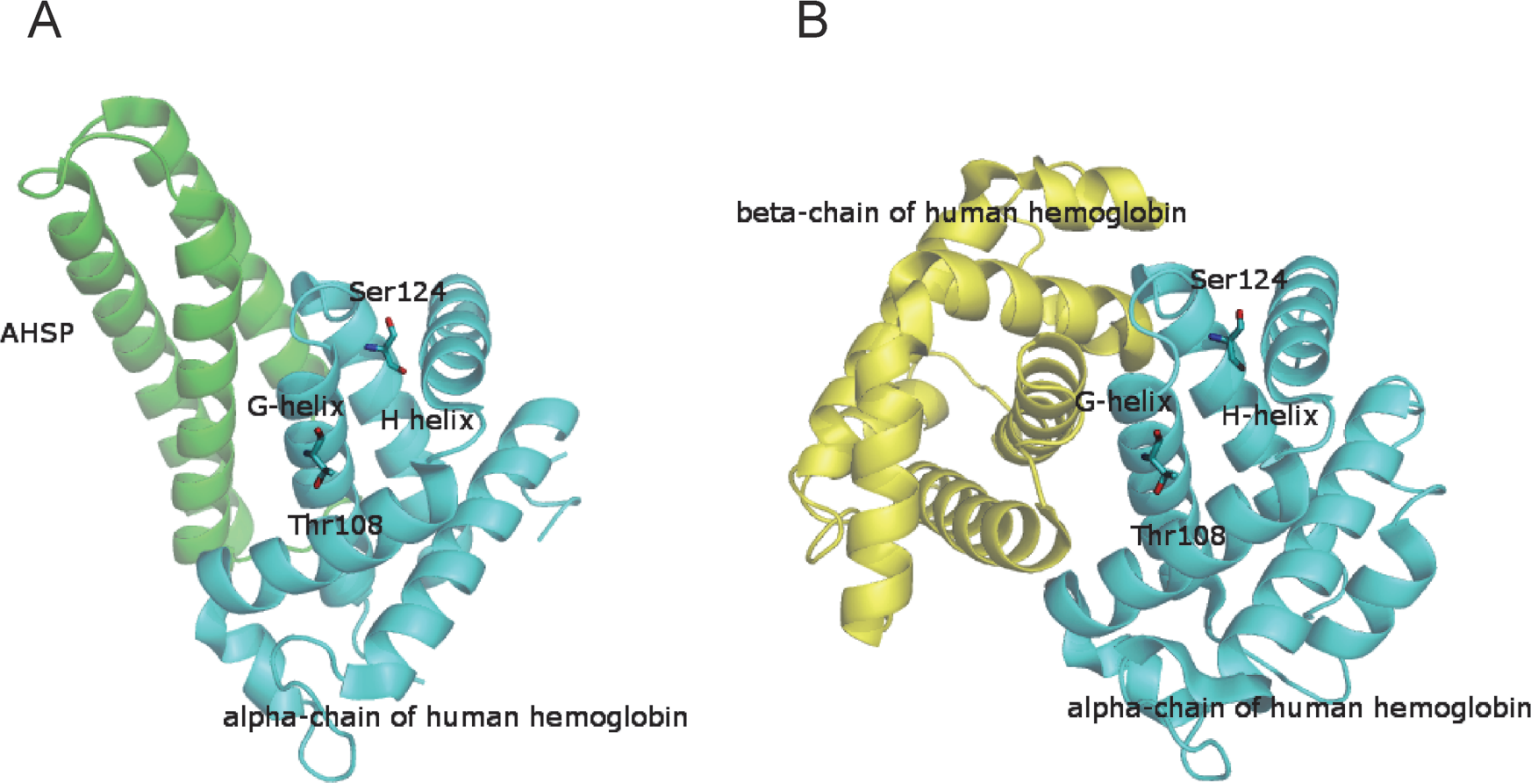
Location of residues discussed in the manuscript on the overall structure of the complex between the α-chain and AHSP and on the structure of the αβ dimer. **A:** Cartoon representation of the three-dimensional structure of the complex between the α-chain and AHSP (PDB code 1Y01) [3]. The α-chain of HbA is highlighted in cyan, whereas the AHSP molecule is in green. In this structure the position of residues 1, 74, 81–91 and 140–142 in the α-globin chain of HbA has not been determined. For this reason the structural representation lacks these residues. **B:** Cartoon representation of the three-dimensional structure of the αβ dimer from the structure of the tetrameric human deoxy hemoglobin (PDB code 2HHB) [23]. α- and β-chains are highlighted in cyan and yellow, respectively.

We carried out an epidemiological project on the molecular basis of α-thalassemia in southern Italy and identified two new variants in the third exon of the α-globin gene: the Hb Rogliano, α1 cod 108 ACC>AAC or α1[α108(G15)ThrAsn] and the Hb Policoro, α2 cod 124 TCC>CCC or α2 [α124(H7)Ser→Pro] [10,11]. These mutations occurred in the G and H helices of the α-globin respectively both involved in the specific recognition of AHSP (Fig. 1A) and β1 chain (Fig. 1B) through hydrophobic interfaces. Molecular characterization of mRNA, globin chain analyses and molecular modelling studies demonstrated that the α-thalassemia defect associated with the two Hb variants originated by different protein instability problems. Hb Rogliano showed an intrinsic instability of the tetramer due to anomalous intra- and inter-chain interactions whereas the mutation occurring in Hb Policoro affected both the amount of variant mRNA and the α-globin-AHSP binding interface. These results underline that various different mechanisms might affect Hb stability playing a role in the pathogenesis of α-thalassemia.

## Materials and Methods

The probands and their families were selected by the Thalassemia Centers collaborating in this study among those referred to them for the hematological diagnosis. A special committee of the Ministry for Research approved the study (Decreto n° 250 of 22 June 1999) and two scientists were the supervisors. We obtained written informed consent from participants for the use of blood samples. Hematological parameters, reticulocytes count, isopropanol and heat Hb stability tests were obtained by standard methods [12].

### DNA analysis

DNA was purified from white blood cells by the salting out method [13]. The α-thalassemia deletions were tested by gap-PCR [14]; point mutations were analyzed by multiplex ARMS and DGGE [15–16]. The α1- or α2-globin genes were sequenced from -181 to +884 (α2) and +894 (α1) as previously reported [17]. An ARMS-PCR assay for the definition of the heterozygous Hb Policoro genotype was set up. The analysis of the RFLP RsaI at 5’ of the α2-globin gene (rs2541669) was carried out on PCR fragments. The SNPs α2+14 (HBA2:c.-24C>G) and α2+861 (HBA2:c.565G>A, rs2685121) were determined by DNA sequencing or DGGE analysis [16–17]. RFPL and SNP association with the mutated α-globin allele was assembled by family segregation studies. All the primers are reported in the S1 Table.

### mRNA analysis

Total RNA from reticulocyte-enriched peripheral blood cells and from PBSC differentiated *in vitro* was isolated with Triazol (Life Technologies, New York, NY, USA) [18]. The α-globin genes cDNA was sequenced by RT-PCR and Automated Cycle Sequencing (S1 Table) [19].

#### Detection of anomalous mRNA

cDNA of anomalous length was separated by acrylamide gel. The RT-PCR of the α-globin gene-at low number of cycles (24 cycles) and containing 0.2 μl of P32 α-dCTP- were carried out using the primers A and H reported in S1 Table generating a cDNA amplicon of 226 bp. Ten μl of the RT-PCR was separated on a 6.5% (37.5:1) Acrylamide gel in TEA 1 X Buffer run at 300V for 6 h. The gel was dried on a gel drier, and the intensity of each band was quantified by using a phosphoimager with ImageQuant software (Molecular Dynamics, Sunnyvale, CA, USA) [20].

#### Semi-quantitative analysis of the mRNA by restriction enzyme and DG-DGGE analysis

A semi-quantitative analysis of the mutated/normal cDNA was carried out by BstEII restriction enzyme analysis, for which the mutation Hb Rogliano eliminates the site 5'-G^GTNACC-3'. The DNA and cDNA PCR amplification was carried out at 24 cycles on two normal and one heterozygous subjects and twice on each DNA or cDNA sample respectively with the primers F and G, generating a DNA amplicon of 190 bp, and with the primers A and E, generating a cDNA amplicon of 230 bp. The amplified fragments were restricted according to the manufacturer’s recommendation with 20 IU of BstEII. 100–200 ng of the digested products were size fractionated and the ratio of undigested/digested bands (that is mutated/normal mRNA or DNA) was obtained as previously reported [19].

A protocol for the semi-quantitative analysis of Hb Policoro cDNA using the DG-DGGE separation was set up [16]. Out of five fragments analyzed throughout the DG-DGGE (data not shown) was selected the only one showing a distinct separation of the homoduplex and heteroduplex bands in a heterozygote subjects. The RT-PCR was carried out at low number of cycles (24 cycles) using the primer H for the reserve transcriptase and the primers B and C for the PCR. After the separation on the DG-DGGE a quantitative analysis of the bands was performed by the Kodak software.

### PBSC differentiated in vitro

Blood samples were from two carriers of the Hb Policoro and from the normal parent. Following the manufacturer’s protocols, total mononuclear cells were isolated by Ficoll gradient (Lymphocyte Separation Medium, ICN, Cappel, Aurora, OH); suspended in 500 μl of PBS + 0.6% ACD-A and purified from the residual red blood cells with 3.0 ml of ammonium chloride solution (StemCell, Vancouver, BC, Canada), putted for 10 min in ice, washed twice with PBS 0.6% ACD-A and suspended in Iscove’s modified Dulbecco’s medium, 2% fetal bovine serum at the concentration of 7x10^6^ cells per 1 ml of medium. The cells were suspended in methylcellulose media HSC-CFU lite with Epo (Miltenyi Biotec, Bergisch Gladbach, Germany) at a final plating concentration of 7x10^5^ cells per 1.1 ml of medium, plated in 35 mm dishes and incubated in 5% CO_2_ at 37°C for the subsequent 7–13 days [18].

At the end of the incubation time the cells were recovered from the plate, washed two times with PBS 0.6% ACD-A, suspended in 250 μl of RSB-100 (10 mM Tris:HCl, pH 7.4, 100 mM sodium chloride, 2.5 mM magnesium chloride, and 0.5% Triton X-100) and the cytoplasmic and cellular mRNA was extract as reported by Rodgers [21]. The cellular membrane was broken by passing the solution through a 1 ml insulin syringe three times, the intact nuclei were pelleted with a quick spin and the supernatant containing the cytoplasmic mRNA was transferred to a tube containing 700 μl of TRI REAGENT (Molecular Research Center, Inc. Montgomery Road, Cincinnati, OH). The nuclei pellet was suspended in 250 μl of RSB-100 and 700 μl of TRI REAGENT was added.

### Globin chain analysis

Hemoglobin analysis was carried out by cation exchange high performance liquid chromatography (HPLC) (Variant II System; Bio-Rad Laboratories, USA). Globin chain analysis was also performed by reverse-phase HPLC Variant I (Bio-Rad System) on a Hibar Lichrosphere 100, 5 μm column (Merck KGaA, Germany) according to the manufacturer’s recommendation [22]. The hemolysate from the patient sample was analyzed by liquid chromatography-mass spectrometry (LC/MS) on a Quattro Micro LC-MS system (Waters, U.S.A.) coupled to an HP 1100 HPLC system (Agilent Technologies, USA). Individual globin chains were separated using a narrowbore C4 Jupiter reverse-phase column (250 x 2.1 mm, 300 A) (Phenomenex, USA). Both MALDI-MS and MALDI-MS/MS experiments were carried out on a 4800 plus TOF-TOF instrument (ABI Sciex Framingham MA USA).

### Structural analysis

We evaluated mutation-induced structural alterations by analysing the structure of α-globin chain of human hemoglobin in the complex with AHSP (PDB code 1Y01) and in the tetrameric α2β2 structure (PDB code 2HHB) [3,23]. Modelling of the mutants have been performed using the program SwissPDBViewer [24] and structural analyses have been performed using Coot [25]. The resulting structures passed all stereochemical and geometric checks in PROCHECK [26]. Figures were prepared with Pymol (www.pymol.org).

## Results

### Hb Rogliano

**Fig 2:**
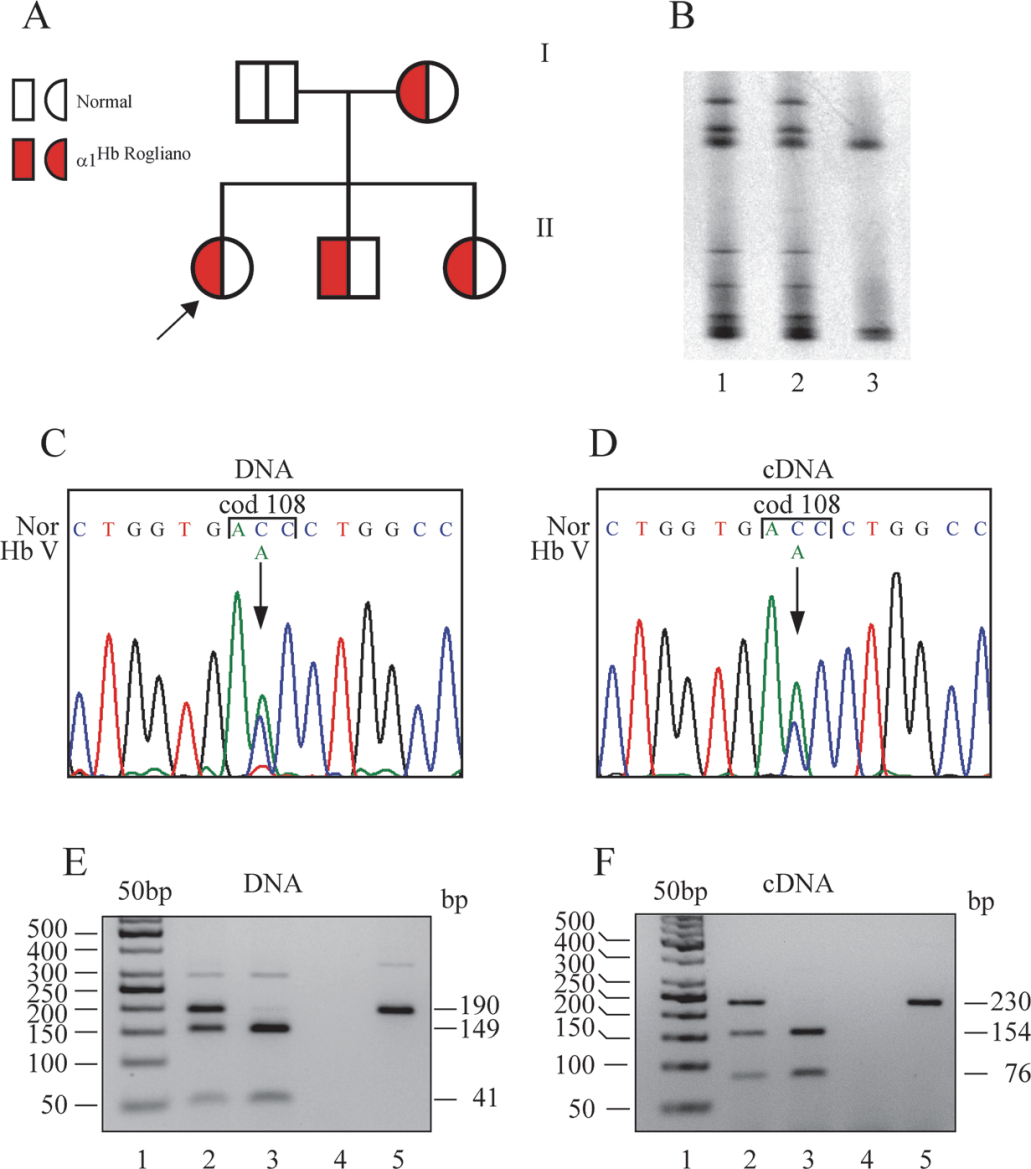
Molecular characterization and cDNA analysis of the Hb Rogliano. **A:** Pedigree of the family. The arrow indicates the proband. **B:** DGGE of the fragment III of the α-globin genes containing the codon 108. Lanes 1 and 2: Hb Rogliano heterozygotes, Lane 3: normal subject. **C** and **D:** DNA and cDNA sequence of the α1-globin gene of the proband from codon 106 to codon 110; the arrow indicates the mutation. **E** and **F:** The fragment of 190 bp (DNA) and 230 bp (cDNA), digested with the restriction enzyme BstEII, and separated on a Nu Sieve 3:1 agarose 4% gel. **E:** Lane 1: 50 bp ladder; Lane 2: DNA of the Hb Rogliano carrier; Lane 3: DNA of the control subject; Lane 4: negative control; Lane 5: undigested sample. **F:** Lane 1: 50 bp ladder; Lane 2: cDNA of the Hb Rogliano carrier; Lane 3: cDNA of the control subject; Lane 4 PCR RT- on the Hb Rogliano carrier; Lane 5: undigested sample. The fragments’ lengths are reported on the left and right.

We identified a new α-globin chain variant in the G Helix at cod 108 of the α1-globin gene named Hb Rogliano or HBA1:c.326 C>A, 108(G15)Thr→Asn in two apparently unrelated families for a total of 7 patients originating from two small towns, Rogliano and Castrolibero, in Calabria, southern Italy [10]. The probands (II-2 Family A and II-1 Family B) (Fig. 2A, Table 1) did not show any clinical symptoms and had been sent to us to evaluate their mild microcytic red blood cell alterations. All seven carriers had low borderline MCV (76–82 fL) and MCH (25.8–27.6 pg) values, associated with increased Simmel and mild RBC morphological alterations. Reticulocytes, serum iron, indirect bilirubin and haptoglobin were within the normal range in all the examined carriers. No Hb variant was detected by cation exchange HPLC; the Hb A2 was normal (Table 1). The presence of the most common α-thalassemia deletions and point mutations was excluded. DG-DGGE analysis identified an anomalous pattern in the third exon of the α-globin genes (Fig. 2B); its sequencing revealed an ACC>AAC substitution at codon 108 of the α1-globin gene (Fig. 2C); no other mutations were detected in either α1- or α2-globin genes. Instability test on fresh blood could not be performed, but the analysis at the IGB, after shipping, was normal.

**Table 1:** Hematologic, biochemical data, α-genotype and SNP analysis of the members of the family with the new Hb Rogliano or α1 cod 108 ACC>AAC.

Family	A				B					Normal ranges
*Parameters*	I-1	I-2	II-1	II-2	I-1	I-2	II-1	II-2	II-3	
Age (years)/sex	52/M	50/F	23/F	18/M	63/M	56/F	30/F	28/M	27/F	
RBC (10^12^/L)	5.11	5.18	4.76	5.94	4.63	5.53	5.07	5.16	4.5	3.58–6.0
Hb (g/dL)	14.1	14.3	12.4	15.3	14.6	14.7	13.4	13.7	12.3	13.0–17.5
Ht (L/L)	41.6	42.6	37.5	45.5	41.5	45.3	40.3	40.9	36.6	42.0–54.0
MCV (fL)	81.4	82.2	78.8	76.6	89.6	81.9	79.5	79.3	81.3	82.0–98.0
MCH (pg)	27.6	27.6	26.1	25.8	31.5	26.6	26.4	26.6	27.3	27.0–32.0
MCHC (%)	33.9	33.6	33.1	33.6	35.2	32.5	33.3	33.5	33.6	32.0–37.0
Ret (%)	1.74	1.74	0.98	1.2	nt	nt	nt	nt	nt	0.5–1.5
Erythrocyte morphology	A	N	A;H	A;H	nt	N	N	A;E;T;H	A;P;H	
Hb A2 (%)	2.7	2.4	2.8	2.6	2.73	2.3	2.2	2.2	2.8	2.0–3.2
Hb F (%)	0.7	0.9	0.8	0.8	0.16	0.9	0.6	0.8	1.3	<1
Serum iron (μg/dL)	61	60	nt	98	129	69	112	98	76	28–170
Bilir tot (mg/dL)	0.5	0.5	0.5	0.6	0.6	nt	nt	nt	nt	0.4–1.20
Bil dir (mg/dL)	0.1	0.2	0	0.1	0.16	nt	nt	nt	nt	0.0–0.5
LDH (U/L)	377	415	360	440	nt	nt	nt	nt	nt	266–500
Haptoglobin (mg/dL)	116	113	165	81	nt	nt	nt	nt	nt	40–240
SIMMEL (40%)	80%	97%	80%	80%	nt	85%	75%	70%	nt	97–100
Isopropanol test	---	---	---	---	nt	+--	+--	+--	nt	
Heat stability test	---	---	---	---	nt	+--	+--	+--	nt	
β-globin chains %	42.10	41.80	42.50	40.80	nt	40.90	40.70	37.80	nt	
α-variant chains %	3.40	0.00	3.50	4.50	nt	4.70	5.20	4.40	nt	
α-globin chains %	39.80	42.90	40.20	39.90	nt	40.20	39.80	40.30	nt	
Rsa I 3’ α1 rs2541669	nt	+/-	nt	+/+	-/-	+/-	+/-	nt	nt	
α2 +14 C>G	nt	+/-	nt	-/-	-/-	-/-	-/-	nt	nt	
α2 +861 G>A rs2685121	nt	-/-	nt	-/-	+/-	-/-	-/-	nt	nt	
α2α1/α2α1^HbRogliano^	yes	N	yes	yes	N	yes	yes	yes	yes	

*Ret*: *Reticolocytes; nt*: *not tested;A*: *Anisocytosis; P*: *Poikilocytosis; H*: *Hypochromia; E*: *Elliptocytosis; T*: *Target cells; N*: *normal*.

To define the molecular mechanism originating the α-thalassemia phenotype three aspects were investigated: a) the relative amount of anomalous mRNA to verify if the mutation induces a known or unknown pathway of mRNA degradation [27–28]; b) the instability and the relative concentration of Hb Rogliano using different diagnostic procedures [29]; c) molecular modelling approaches were used to predict the interaction of the α-chain variant with both the β1-globin chain and AHSP.

Sequencing of the α1-globin gene cDNA revealed the presence of mutated cDNA (Fig. 2D); α1 cDNA analysis with BstEII displayed two bands in normal subjects (154 bp and 76 bp) and one additional undigested band in the carrier (230 bp); the same pattern was also detected in the DNA samples (Fig. 2E-F). Semi-quantitative analysis indicated that the amount of variant mRNA was comparable to the normal mRNA (mutated:normal cDNA = 48:52).

**Fig 3:**
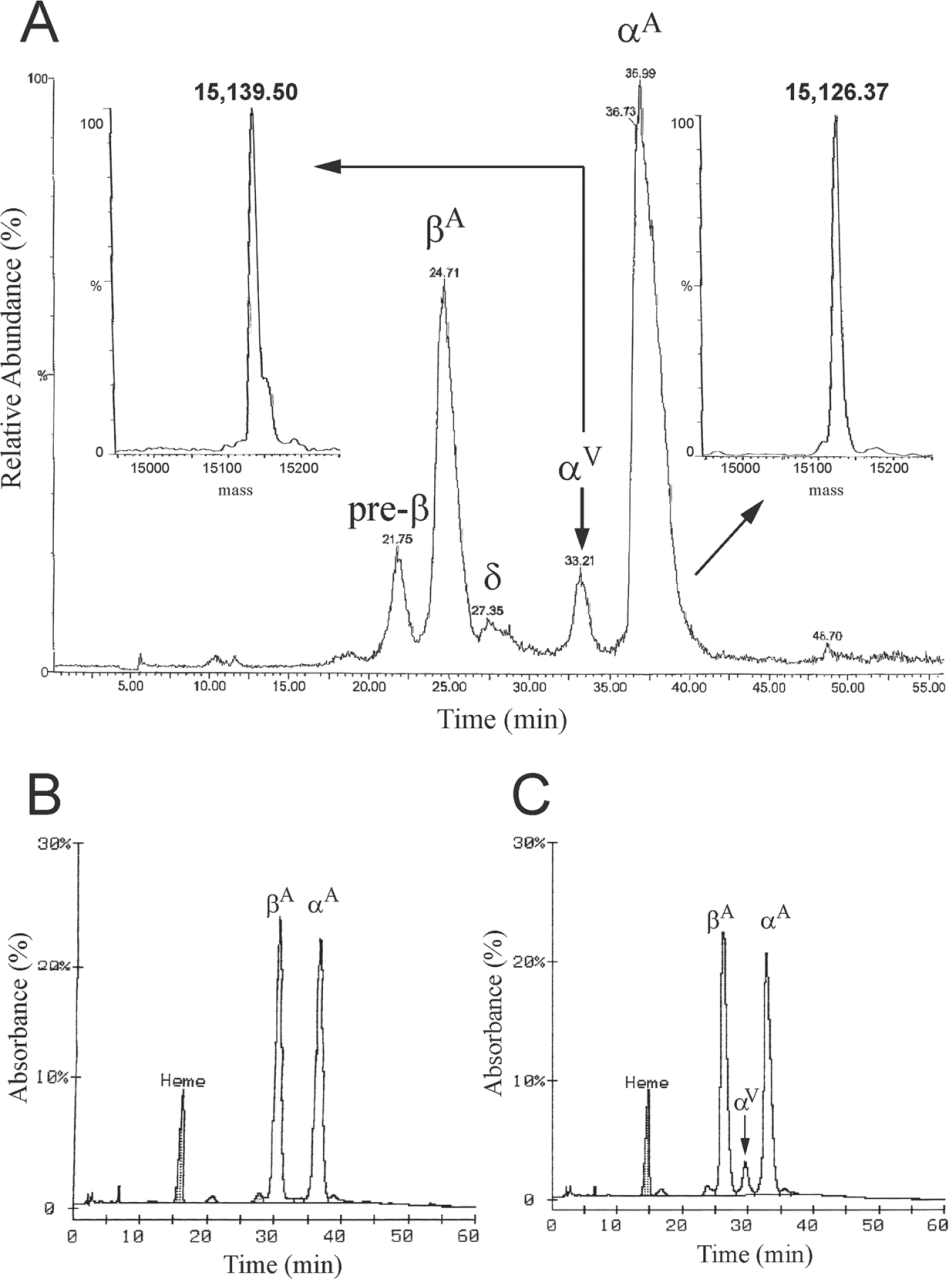
Liquid chromatography-mass spectrometry analysis (LC/MS) of the hemolysate from a Hb Rogliano carrier. **A**: Total ion current (TIC) of the LC-MS analysis of the globin chains. The anomalous globin chain eluting before the normal α-globin is marked as α^V^. The arrows indicated the electrospray mass spectra of the variant and the normal α-globin chains. **B** and **C:** Reverse-phase HPLC separation of globin chains. **B**: normal control; **C**: Hb Rogliano carrier. The variant α-globin chain (α^V^) is indicated by an arrow.

The relative amount of the variant globin of Hb Rogliano was first evaluated by LC-MS analysis of the hemolysate. The electrospray mass spectrum revealed the presence of a variant α-globin chain with a molecular mass of 15,139.500.4 Da, about 13 Da higher than the normal α-globin chain (MW 15,126.37 Da), the expected mass change due to the Thr→Asn substitution (Fig. 3A). This anomalous peak accounted for about 6% of the total globins. MALDI-MS analysis of the corresponding tryptic digest confirmed the presence of an anomalous 100–127 peptide peak (+ 13 Da) and the tandem MALDI-MS analysis identified the occurrence of the Thr→Asn amino acid substitution at position 108 (data not shown). No other post-translational modifications were detected.

RP-HPLC analysis was then carried out on fresh hemolysed blood samples demonstrating that the α-globin chain variant was present in all carriers ranging from 3.40 to 5.20%, showing a retention time lower (29.53 min) than the normal α-chain (32.76 min) (Fig. 3B-C, Table 1).

A structural analysis by molecular modeling approaches was then performed. Homology modeling has been often used to unveil the effect of single point mutations on a protein structure [30–31], even for HbA [32], although a long-time scale MD simulation is need to study in detail possible subtle structural or dynamic modifications induced by mutations.

**Fig 4:**
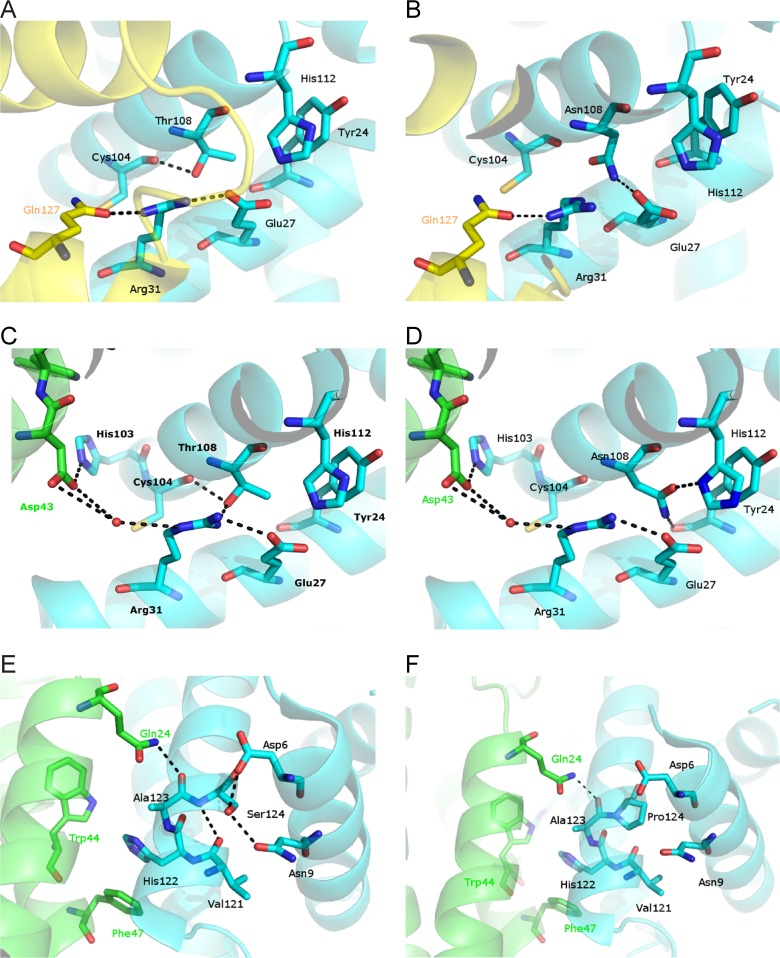
Environment of Thr/Asn108α and Ser/Pro124α in the structure of the human hemoglobin tetramer and of AHSP-alpha globin chain complex structure. **A:** Structural representation of the environment of Thr108α in the structure of the human hemoglobin tetramer. **B:** Structural representation of the environment of Asn108α in the plausible structure of human hemoglobin T108N mutant. In panels A and B, the α- and β-chains are coloured in cyan and yellow, respectively. **C:** Structural representation of the environment of Thr108α in the structure of the complex between AHSP and the α-chain of human hemoglobin. **D:** Structural representation of the environment of Asn108α in the structure of the plausible hypothetical complex between AHSP and the α-chain variant of human hemoglobin. There are no major differences in side chain orientation between Fig. 4C and 4D if one exclude the flipping of the His112α imidazole ring. **E:** Structural representation of the environment of Ser124α in the structure of the complex between AHSP and the α-chain of human hemoglobin. **F:** Structural representation of the environment of Pro124α in the structure of the plausible hypothetical complex between AHSP and the α-chain variant of human hemoglobin. In panels C-E, the α-chain of HbA is coloured in cyan, whereas AHSP is in green. In all the panels, the nitrogens are coloured in blue, oxygens in red and hydrogen bonds are indicated as dashed lines.

In the structure of human hemoglobin, Thr108α is buried and close to the α1β1 interface (Fig. 4A) [23]. In particular, in the X-ray structure of human deoxyhemoglobin (PDB code 2HHB), the side chain of the Thr makes an hydrogen bond with the carbonyl oxygen of Cys104α (Fig. 4A). The Thr108α side chain is located close to Arg31α side chain (distance 3.4 Å) which is involved in the formation of a stabilizing hydrogen bonds with the side chain of Glu27α and with the side chain of Gln127β. The substitution of the Thr108α with an Asn residue could significantly affect the structural feature of this region, modifying the interaction between the Arg31α and the Gln127β. Indeed, in the mutated protein, the Asn side chain could form an hydrogen bond with Glu27α side chain, thus disrupting the pattern of hydrogen bonds that fix the Arg31α side chain in the right position to interact with Gln127β (Fig. 4B).

In Fig. 4 (panels C and E) the X-ray structure of AHSP bound to Fe(II) α-globin chain of human hemoglobin is shown. The Thr side chain makes a hydrogen bond with the carbonyl oxygen of Cys104α and with the NH atom of Arg31α, which is hydrogen bonded to a water molecule that in turn is in contact with the OD atoms of Asp43 of AHSP [3]. Therefore, the replacement of Thr108α with Asn may not alter the AHSP/α-chain interaction, since the residues around position 104 are not involved directly in the recognition process (Fig. 4C). In this respect, it is interesting to note that the Asn108α side chain might well be accommodated in the environment of position 104 where it could also form a hydrogen bond with the side chain of His112α (Fig. 4D).

Data from the structural analysis are in agreement with the observation that, in the case of this mutation, the overall interactions between αHb and AHSP are suboptimal compared to those of the α1-β1 complex [3].

All these results suggest that the variant chain is normally synthesized and complexed with AHSP; nevertheless, the interaction with the β-globin chain causes instability and precipitation of Hb Rogliano, leading to a mild α-thal phenotype and morphological alteration in the RBC, without hemolysis as shown by the normal bilirubin and LDH value (Table 1).

Identification of the cod 108 mutant ACC>AAC on both the α1- and α2-globin genes could be explained either by gene conversion mechanism or independent mutation [10,33]. The first hypothesis implies that the gene conversion had occurred in the region II of homology (171 bp long) [34]. Considering that Hb Bleuland (HBA2:c.326C>A), bearing the same mutation in the α2-globin gene, shows a low frequency in the Mediterranean area, that Hb Rogliano (HBA1:c.326C>A) by sequencing shows the α1-globin structure and the short length of region II, the probability of gene conversion is very low. More probably, Hb Rogliano originated from an independent mutation.

To study the origin and spreading of the variant allele we analyzed three non-pathogenic sequence variations very close to or inside the α-globin genes, showing characteristics useful for the genetic analysis of populations [16,34]. Identification of the same haplotype “+ - -” in both families indicates that very likely the origin of Hb Rogliano is unique (Table 1).

### Hb Policoro

Hb Policoro was identified in five unrelated families in a total of 12 carriers. Two families were originating from Basilicata, two from Campania and one from Sicily, all regions of Southern Italy. The variant was first identified in a family from Policoro and was then named Hb Policoro [11]. In all families the probands showed mild microcythemia (MCV 74–78; MCH 25–27) with normal iron metabolism, Hb A2 in the normal range and no Hb variants were observed by cation-exchange HPLC or cellulose acetate electrophoresis (Table 2). 3/5 families were sent to us for the suspect of presence of unstable hemoglobin due to the occurrence of inclusion bodies and positive isopropanol instability test (Table 2).

**Table 2:** Hematologic, biochemical data and α-genotype of the families with the new Hb Policoro or α2 cod 124 TCC>CCC.

Family	C				D	E					F				G			
*Parameters*	I.1	I.2	II.1	II.2	I.1	I.1	I.2	II.1	II.2	II.3	I.1	I.2	II.1	II.2	I.1	I.2	II.1	II.2
Age (years)/sex	45/M	40/F	19/F	17/F	14/M	39/M	38/F	19/F	18/M	10/M	66/M	55/F	19/M	17/M	62/M	58/F	29/F	26/F
RBC (10^12^/L)	5.44	4.42	4.85	4.80	5.35	5.30	5.14	4.77	5.29	5.20	5.59	4.72	4.92	5.38	5.50	5.45	5.07	4.70
Hb (g/dL)	15.0	13.9	12.2	12.1	13.4	16.7	14.5	11.7	16.7	13.4	17.1	12.8	13.2	14.0	15.1	15.0	11.8	14.2
Ht (L/L)	45.2	41.8	37.8	37.5	40.5	48.2	42.2	35.5	47.9	38.6	47.9	36.7	38.1	40.2	45.8	46.5	35.7	42.9
MCV (fL)	83.1	94.6	77.9	78.1	75.7	91.0	82.0	74.0	90.0	74.0	86.0	78.0	77.0	75.0	83.0	85.0	70.0	91.0
MCH (pg)	27.6	31.4	25.2	25.2	25.0	31.5	28.1	24.6	31.5	25.8	30.5	27.1	26.8	26.0	27.6	27.5	23.3	30.3
MCHC (%)	33.2	33.3	32.3	32.3	33.1	34.7	34.3	33.0	34.9	34.7	35.6	34.9	34.7	34.8	33.0	32.1	33.1	33.2
Serum iron (μg/dL)	164	122	72	71	nt	nt	nt	nt	nt	nt	nt	nt	nt	113	nt	nt	44	nt
Ferritin (ng/ml)	185.7	26.5	42.3	32.0	nt	nt	nt	nt	nt	nt	nt	nt	nt	67.6	nt	nt	97.0	nt
ZPP (μg/dL)	24	24	19	22	27	29	30	37	27	27	nt	nt	nt	nt	nt	nt	nt	nt
Bilir tot (mg/dL)	nt	nt	nt	nt	nt	nt	nt	nt	nt	nt	0.82	0.47	0.79	0.34	nt	nt	nt	nt
Bil dir (mg/dL)	nt	nt	nt	nt	nt	nt	nt	nt	nt	nt	0.12	0.04	0.11	nt	nt	nt	nt	nt
Hb A2 (%)	2.8	3.2	3.1	2.8	3.1	2.7	2.6	2.7	2.7	2.9	2.5	2.5	2.7	2.9	3.1	1.8	3.0	2.9
Hb F (%)	nt	0.2	nt	0.2	1.0	0.8	1.8	1.3	0.4	0.8	0.6	<1	<1	0.4	0.9	0.9	0.5	0
Electro/ HPLC Hb	nt	nt	nt	N	nt	nt	nt	nt	nt	nt	N	N	N	N	nt	nt	N	nt
Ret (%)	nt	nt	nt	nt	nt	nt	nt	nt	nt	nt	1.0	1.7	1.3	2.0	0.7	1.6	1.8	nt
I.B.	nt	nt	nt	nt	nt	nt	nt	Pos	nt	Rare	nt	nt	nt	nt	Abs	Abs	Pos	Abs
Isopropanol test	nt	nt	nt	nt	nt	nt	nt	nt	nt	nt	Neg	Pos	Pos	nt	Pos	Neg	Pos	Neg
Rsa I 3’ α1 rs2541669	-/-	+/-	+/-	nt	-/-	-/-	+/-	-/-	nt	nt	+/-	-/-	nt	+/-	+/-	nt	-/	nt
α2 +14 C>G	nt	nt	-/-	nt	-/-	+/-	-/-	-/-	+/-	+/-	-/-	-/-	nt	-/-	nt	nt	-/	nt
α2 +861 G>A rs2685121	nt	nt	+/-	nt	+/-	+/-	+/-	+/+	-/-	+/-	-/-	+/-	nt	+/-	nt	nt	+/	nt
α^Hb Pol^ α α α	yes	N	yes	yes	yes	N	yes	yes	N	yes	N	yes	yes	yes	yes	-α^3.7^/αα	yes/-α^3.7^	N

*Ret*: *Reticolocytes; I*.*B*.: *Inclusion Body; N*: *normal; Hb Pol*: *Hb Policoro; Pos*: *Positive; Abs*: *Absent; Neg*: *Negative; nt*: *not tested*.

Besides the carriers, one patient was compound heterozygote for the α2 cod 124 TCC>CCC mutation and the -α ^3.7^ deletion. This patient showed a more severe reduction of Hb (11.8 g/dL) and MCV (70 fL), presence of inclusion bodies and was positive to the instability test.

**Fig 5:**
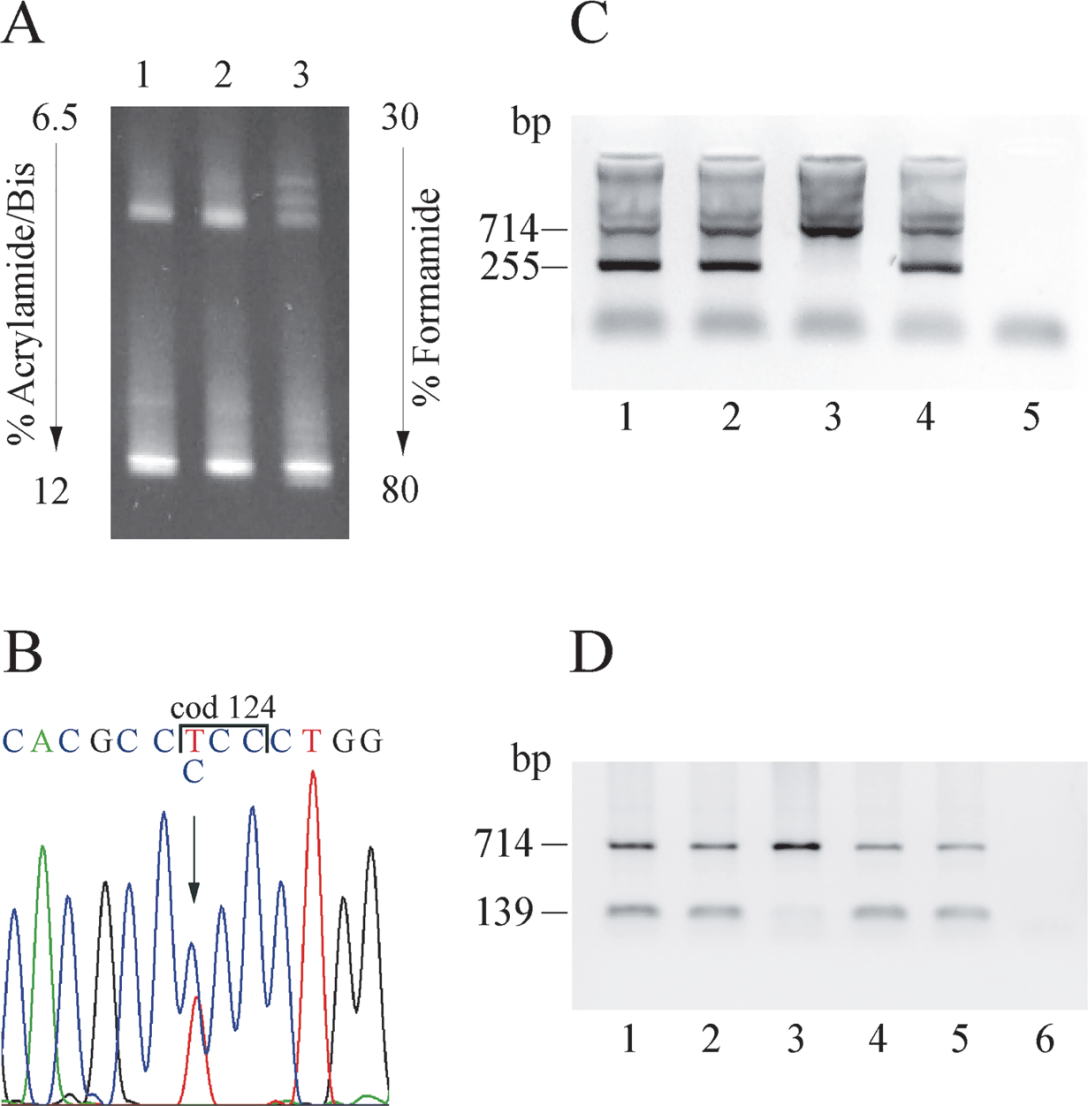
Molecular characterization of the Hb Policoro. **A:** DGGE of the fragment III of the α-globin genes containing the codon 124. Lanes 1 and 2: normal subjects, Lane 3: Hb Policoro heterozygote. **B:** DNA sequence of the α2-globin gene of the proband from codon 122 to codon 126; the arrow indicates the mutation. **C:** ARMS for the screening of carriers for Hb Policoro: the control amplicon was of 714 bp, the amplicon specific for the mutation was 255 bp long. Lanes 1, 2, 4: Hb Policoro heterozygotes; Lane 3: normal subject; lane 5: negative control. **D:** ARMS with the normal primer at codon 124 for the genotyping of the Hb Policoro carriers: the control amplicon was of 714 bp, the amplicon specific for the cod 124 normal allele was 139 bp long. Lanes 1, 2: Hb Policoro heterozygotes; Lane 3: compound heterozygote for the Hb Policoro and the -α^3.7^ deletion; Lane 4:-α^3.7^ deletion heterozygote; Lane 5: normal subject; Lane 6: negative control. The ARMS-PCR conditions were: hot start 95° for 10'; PCR: 94° for 45'', 63° for 45'', 72° for 45'', for 30 cycles.

The screening for the most common α-thalassemia deletions (-α^3.7^,-α ^4.2^,—^MED^,-(α)^20.5^) and point mutations was negative. DG-DGGE analysis identified an anomalous pattern in the third exon of the α2 globin gene (Fig. 5A); sequence analysis showed the presence of the mutation α2 cod 124 TCC>CCC in 3 carriers (Fig. 5B) while no other mutations were detected in both the α1 and α2 globin genes. The new variant called Hb Policoro or α2 [α124(H7)Ser→Pro] is the second identified at codon 124 of the α-globin gene. Hb Batley (α1 or α2 124(H7) Ser→Phe), bearing a mutation at the same position, was associated to normal hematological phenotype and the variant hemoglobin comigrated with Hb A2. No data are available on the amount of Hb Batley and on the association with α1 or α2 because the DNA sequence changes was computed [35].

**Fig 6:**
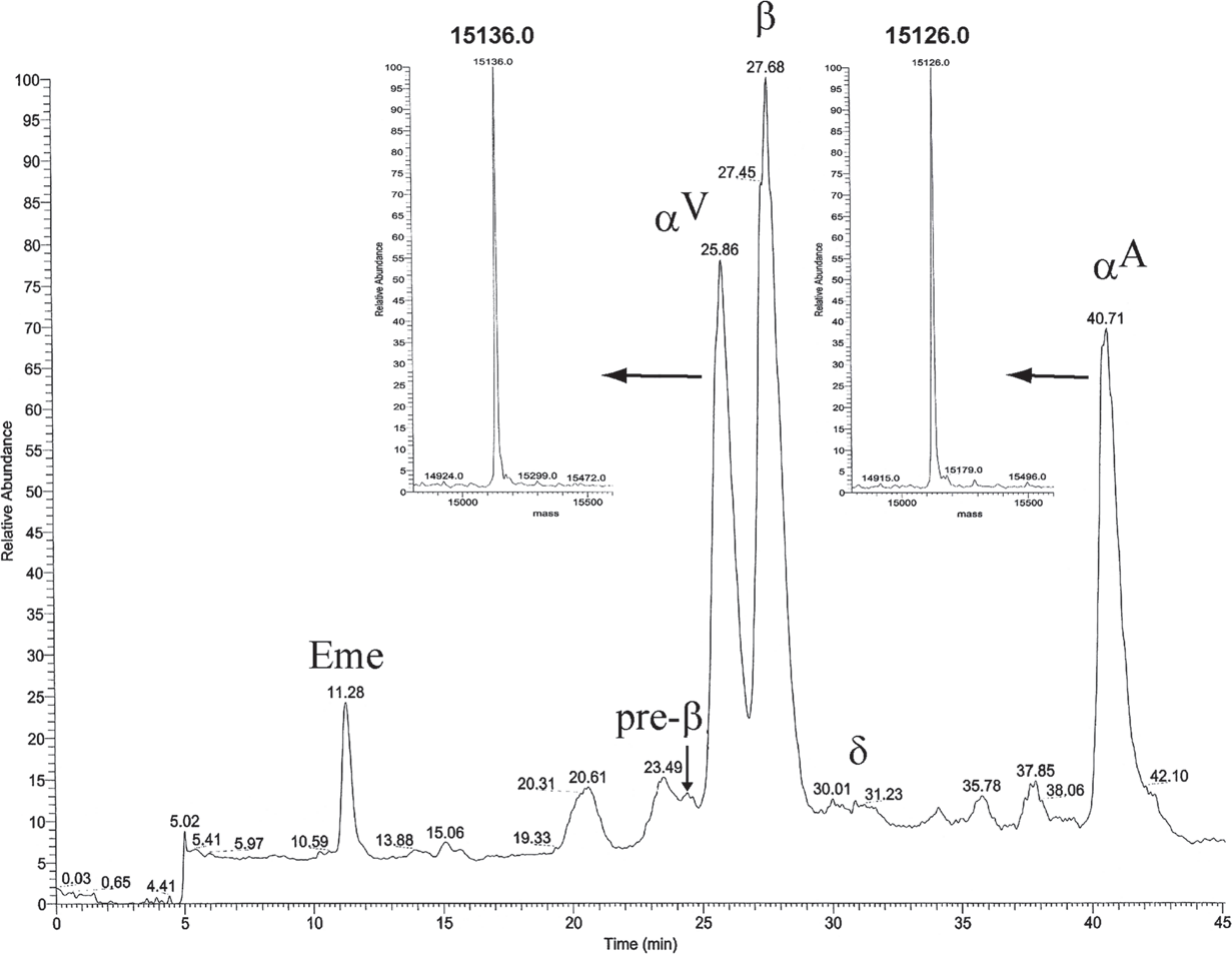
Total ion current (TIC) of the LC-MS analysis and electrospray mass spectra of variant and normal α-chains. Liquid chromatography-mass spectrometry analysis (LC/MS) of globin chains precipitated from the hemolysate of a Hb Policoro carrier after a 20 minutes of 17% isopropanol incubation. The anomalous globin chain eluting before the normal β-globin is marked as α^V^.

To investigate the molecular mechanism originating the α-thalassemia phenotype associated with Hb Policoro, we examined the variant both at the protein and mRNA level. Globin chains, purified from fresh hemolysed blood and from the centrifuged sediment after 20 min isopropanol incubation, were analyzed by LC-MS/MS. The electrospray mass spectrum revealed the presence of a variant α-globin chain only in the sediment from the isopropanol test showing a molecular mass of 15,136.0 Da, about 10 Da higher than the normal α-globin chain (MW 15,126.0 Da), the expected mass change due to the Ser→Pro substitution (Fig. 6). The variant peak was absent in the fresh hemolysed blood of the carriers. The absence of the variant in the peripheral blood is a characteristic common to others unstable variants [1].

**Fig 7:**
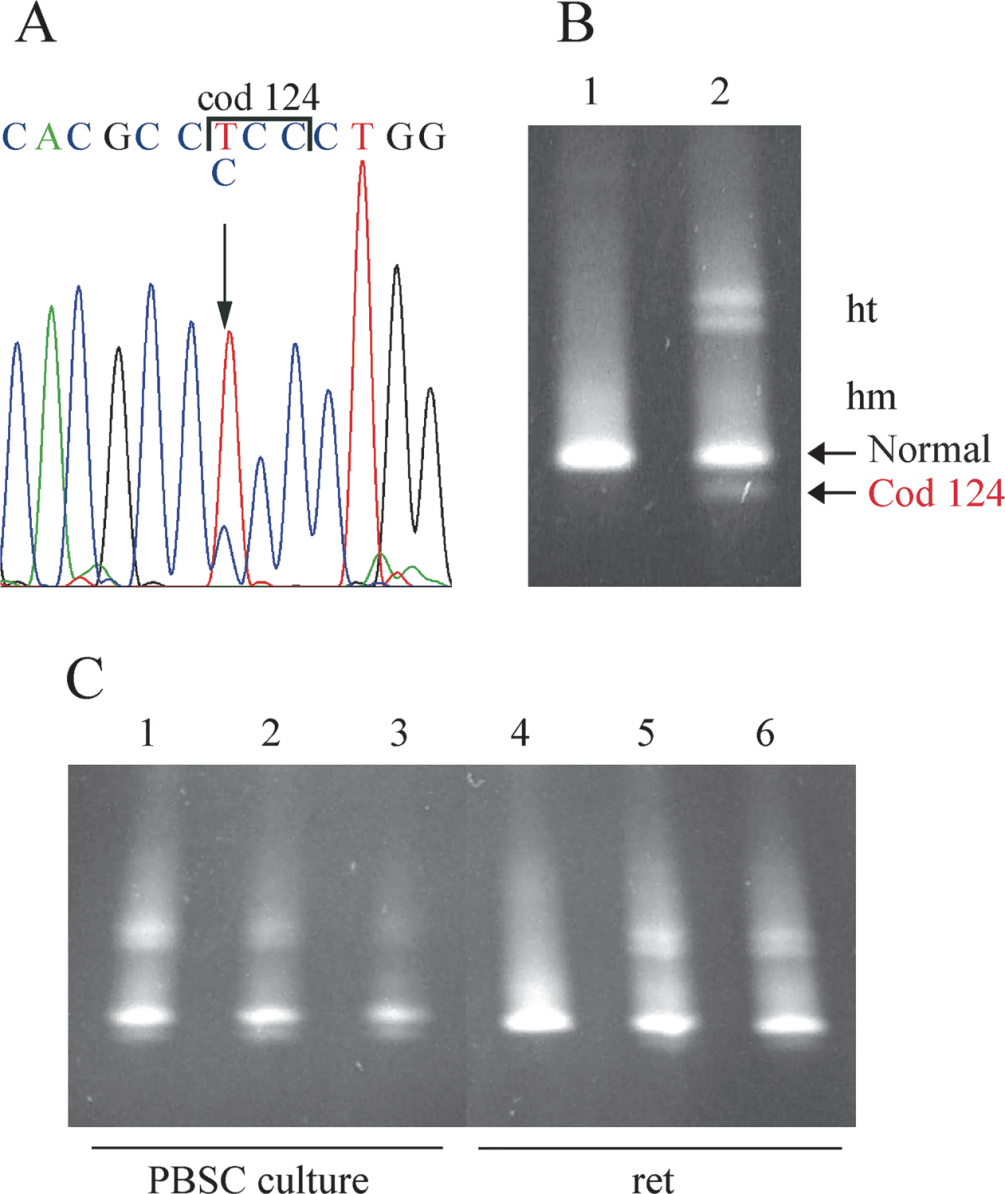
cDNA analysis of the Hb Policoro. **A:** cDNA sequence of the α2-globin gene of the proband from codon 122 to codon 126; the arrow indicates the mutation. **B:** DGGE of the cDNA fragment containing the exon three of the α2-globin gene. Lanes 1: normal subject, Lane 2: Hb Policoro heterozygote. **C:** DGGE of the cDNA fragment containing the exon three of the α2-globin gene. Lanes 1, 2, 3: total (1), nuclear (2) and cytoplasmic (3) cDNA from PBSC at 11 day of culture of a Hb Policoro heterozygote. Lanes 4: normal subject, Lane 5, 6: cDNA from reticolocytes of Hb Policoro heterozygote.

The relative amount of the mutant cDNA was evaluated to verify if the mutation induced mRNA degradation. Sequencing of the α2 globin gene cDNA revealed the presence of the mutated cDNA (Fig. 7A). The anomalous peak C was smaller than the normal T suggesting a reduction of the mutant mRNA. This assumption was investigated by different approaches.

Following DG-DGGE separation, a semi-quantitative analysis of the homoduplex and heteroduplex cDNA bands was carried out to evaluate the level of mutant cDNA. As shown in Fig. 7B, the four bands had different intensity. The homoduplex mutant was about 1/6 of the normal homoduplex, whereas the heteroduplex bands showed comparable intensity within each other but lower than the normal homoduplex. Semi-quantitative analysis was performed as follows: amount of Hb Policoro cDNA = Mutant Homoduplex + (sum of the two heteroduplex bands/2); amount of normal cDNA = Normal Homoduplex + (sum of the two heredoduplex bands/2). Analysis of the data by KodaK software indicated that the amount of Hb Policoro cDNA in the reticulocytes from peripheral blood was about 25% of the normal globin chain mRNA (Fig. 7B).

Reduction of the cDNA could be explained by the occurrence of a cryptic splicing site activated by the mutation. Analysis of Hb Policoro with software for the prediction of alternative splicing site gave negative results. This prediction was confirmed by the analysis on acrylamide gel of full length α-globin cDNA (data not showed) and of an ExII-ExIII cDNA fragment amplified using P32 dCTP revealing the absence of anomalous bands and aberrant splicing (S1 Fig.).

Finally, we investigated whether the mRNA degradation occurred in the nucleus or cytoplasm by determining the amount of mRNA in the different compartments. PBSC were differentiated *in vitro* for eleven days and the mRNA was extracted from nucleus, cytoplasm and total cells [21]. Semi-quantitative analysis of cDNA from PBSC showed that in the three fractions the amount of mutant cDNA was comparable being respectively 29% in the nucleus, 27% in the cytoplasm and 30% in the total cells. These values suggested that the mutant mRNA was rapidly degraded in the nucleus (Fig. 7C) but showed normal stability when transferred to the cytoplasm.

The instability of Hb Policoro indicated by the isopropanol stability test was investigated by molecular modeling approaches. In the structure of human hemoglobin, the Serine at codon 124 is located on the H helix (Fig. 1); it interacts with Asp6α, Asn9α and Ala123α residues (Fig. 4E). In particular, in the X-ray structure of human deoxy hemoglobin (PDB code 2HHB), the Ser side chain forms a hydrogen bond with the carboxylate OD1 atom of Asp6α and the ND2 atom of Asn9α (Fig. 4E). The OG atom is also in contact with the carbonyl oxygen of Ala123α and with water molecules. In turn the carbonyl oxygen of Ala123α interacts with AHSP Gln24. Substitution of Ser124α with Pro could significantly modify the structural features of this region, since, as it is well known, the Pro residue destabilizes the alpha helical conformation [36]. Indeed, it is likely that the substitution S124P might lead to a loss of a number of hydrogen bonding interactions stabilizing this region (Fig. 4F). On this basis, it could be expected that the substitution Ser124Pro could alter the overall conformation of the α-chain and its interaction with AHSP. Indeed, the mutation site is close to the α-chain/AHSP interface (Figs. 1 and 4E).

Thus far 8 of the 21 amino acid residues comprising the H Helix of the α-globin chain have been replaced by Proline [35]. Six of these variants (Hb Voreppe, Hb Quong Sze, Hb Sun Prarie, Hb Bibba, Hb Verona, Hb Attleboro) are unstable causing α-thalassemia phenotypes with two of them (Hb Utrecht and Hb Questembert) being very unstable and originating severe hemolytic anemia with an α-thalassemia like phenotype.

To study the origin and spreading of the variant allele we analyzed three non-pathogenic sequence variations very close to or inside the α-globin genes, showing characteristics useful for the genetic analysis of populations [16,34]. Identification of the same haplotype RsaI (-) +14 (-) e +862 (+) “-−+” in all families (Table 2) indicated that most probably the origin of Hb Policoro is unique.

Two single-ARMS protocols were developed for the direct identification and to define the heterozygous/homozygous genotype for the Hb Policoro. The mutant ARMS protocol was used to confirm the presence of the mutation in patients showing the peculiar anomalous DG-DGGE pattern (Fig. 5C) and in the relatives of the carriers identified by direct sequencing. The normal cod 124 ARMS protocol was set up on the only carrier of a deletion showing the genotype α^Hb Pol^αPol/-α^3.7^ (Fig. 5D). The relatively wide distribution of Hb Policoro, observed in three regions of Southern Italy, suggested that the presence of this variant should be tested in patients from Italy (and also from Mediterranean area) with α-thalassemia phenotype and absence of the most common mutations.

## Discussion

Multiple diagnostic approaches were used to elucidate the mechanisms of α-thalassemia phenotype induced by two new hemoglobin variants, Hb Rogliano and Hb Policoro. In the case of Hb Rogliano, semi-quantitative analysis of variant mRNA ruled out the occurrence of any degradation process generating a reduction in the mutated cDNA. Analysis of the variant chain in the peripheral blood indicated Hb instability as the *in vivo* mechanism involved in the onset of the α-thalassemia phenotype.

Proteolytic degradation of unstable α-globin variant Hbs originating the α-thalassemia defect might be related to either an intrinsic instability of the tetramer or an impaired binding to the α-AHSP chaperone. Inspection of HbA tetramer and AHSP-alpha globin complex structures showed that position 108 is not directly involved in interaction with AHSP while it plays an important role in the α1-β1 interface. The Thr→Asn substitution could be associated with the loss of stabilizing interactions leading to an overall instability of the Hb molecule.

Position 108 of the α-globin is conserved among the corresponding orthologs, human, cow, pig, rat, and mouse [4]. The same nucleotide (ACC>AAC) and amino acid (Thr→Asn) substitution identified in Hb Rogliano was described in the α2-globin gene of Hb Bleuland or HBA2:c.326C>A identified in a family of Surinamese-Hindustani origin and in a family from Iran [33,37]. All carriers showed mild α-thalassemia phenotype with borderline microcytic hypochromic parameters (MCV range of 76–80). Separation of hemoglobin on alkaline starch gel electrophoresis, capillary electrophoresis and HPLC revealed normal patterns. No data about the variant chain have been reported [33,37].

To determine the role of the AHSP in the clinical expression of unstable α-Hb variants, Vasseur et al. co-expressed ten α-globin chain variants with the AHSP chaperone in cellular systems [38]. The recovered amount of the GST-AHSP/GST-α-Hb^*Bleuland*^ complex was only slightly decreased (45%) compared to GST-AHSP/GST-α-Hb^WT^ (53%) indicating that in this model system the interaction of the α-globin chain variant with AHSP was normal [38]. The structural analysis of Hb Rogliano confirmed Vasseur's data which showed that the interaction of the α-globin chain of the HbA variant with AHSP is unaffected by the mutation.

The presence of the variant chain further confirmed that the mutation did not affect the interaction with the AHSP chaperone. In that case, a rapid degradation of the variant had to be expected leading to its disappearance from the peripheral blood as reported for Hb Foggia (HBA2:c.353T>C) [19].

Hb Policoro is an example of mutations triggering alteration of multiple processes eventually leading to an α-thalassemia phenotype. The introduction of a Proline residue within the H helix of α-chain immediately pointed out to instability as the mechanism causing the thalassemia defect. However, quantitative analysis of the mRNA revealed a 25% reduction of variant mRNA suggesting that the nucleotide substitution might either activate a degradation process or induce a reduction of mRNA stability. The mechanism leading to the reduction of mRNA appears completely unclear; the sequence analysis of the promoter of the Hb Policoro did not show any point mutation ruling out a decrease in mRNA transcription; the analysis of the mRNA level in the PBSC differentiated *in vitro* indicated that the stability was not altered considering that the % of mutant mRNA was almost the same than in the reticulocytes; the activation of alternative splicing was also excluded.

This is the first experimental evidence that a single nucleotide mutation within the coding region of the α-globin gene affects mRNA expression level and causes an α-thalassemia defect. This data suggest that other regions besides the 3’ UTR may contribute to the degradation or the stabilization of the α-globin mRNA [21]. Since degradation seems to occur in the nucleus, it may be possible that the mutation abolishes the interaction with unknown nuclear factors able to protect the mRNA from degradation. More detailed studies are needed to understand the mechanism behind the reduction of the variant mRNA.

It could be interesting to compare the mRNA level of variant described in close proximity to define if there is a consensus sequence involved in mRNA degradation. Unfortunately, in most of the cases, data on the mutant mRNA level are not reported with the exception of Hb Quong Sze where a normal amount of variant mRNA was described [39]. These data might indicate that only specific mutations are able to affect mRNA level.

Besides the decrease in the mRNA level, the absence of the variant chain in the peripheral blood and its occurrence in the precipitate of the isopropanol stability test strongly suggested that tetrameric instability also contributed to the pathogenesis of α-thalassemia. Indeed, molecular modelling investigations indicated that the presence of Pro124α impaired the interaction of the α-chain with the AHSP chaperone leading to a rapid precipitation of the variant globin to form inclusion bodies.

Among the Hb variants bearing a Pro residue within the H helix, Hb Voreppe (position 123) and Hb Quong Sze (position 125) bear mutations in close proximity to position 124 [39–40]. Both these variants have never been observed in the peripheral blood, although the amount of Hb Quong Sze mRNA was comparable to the normal one. Analysis of Hb Quong Sze indicated that the mutated chain is destroyed in the bone marrow before assembling with the partner subunit, leading to a pure α-thal2 (or α^+^-thal) including some degree of ineffective erytropoiesis [39].

In summary, we demonstrated that the α-thalassemia associated with the two Hb unstable variants, both located in the third exon of the α-globin gene, originated by different defects. Hb Rogliano showed an intrinsic instability of the tetramer due to anomalous intra- and inter-chain interactions suggesting that the variant chain is normally synthesized and complexed with AHSP but rapidly degraded because it is unable to form the α1β1 dimers. On the contrary, in the case of Hb Policoro two different molecular mechanisms were shown to be involved in the onset of the α-thalassemia phenotype, the reduction of the variant mRNA level by an unclear mechanism and the protein instability due to impairment of AHSP interaction.

These data highlighted that multiple approaches including mRNA quantification are needed to clarify the mechanisms leading to the α-thalassemia defect and to define the contribution that the altered process might give to the observed phenotype thus providing important guidance in the diagnosis of unstable variants.

## Supporting Information

S1 Fig Polyacrylamide gel electrophoresis for the detection of anomalous mRNA. (PDF)

S1 Table Oligonucleotides used as Primers in the reported applications. (PDF)
