# Supplementary material for: α-Thalassemia Associated with Hb Instability: A Tale of Two Features. The Case of Hb Rogliano or α1 Cod 108(G15)Thr→Asn and Hb Policoro or α2 Cod 124(H7)Ser→Pro
Source: PLoS One. 2015 Mar 2;10(3):e0115738. doi: 10.1371/journal.pone.0115738 (PMC4346585; doi:10.1371/journal.pone.0115738)
Supplement: S1 Fig — (PDF) [file pone.0115738.s001.pdf]

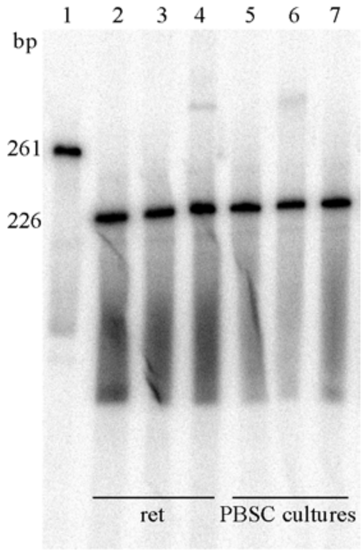

Supplementary Figure S1: Polyacrylamide gel electrophoresis of a cDNA fragment of 226 bp containing the cod 124 of the alpha globin gene. Lane 1: DNA fragment of 261 bp; Lanes 2, 4: cDNA from reticulocytes of two Hb Policoro heterozygotes. Lane 3: cDNA from reticulocytes of a normal subject. Lanes 5, 6, 7: total (5), nuclear (6) and cytoplasmic (7) cDNA from PBSC at 11 day of culture of a Hb Policoro heterozygote.
