# Supplementary material for: α-Thalassemia Associated with Hb Instability: A Tale of Two Features. The Case of Hb Rogliano or α1 Cod 108(G15)Thr→Asn and Hb Policoro or α2 Cod 124(H7)Ser→Pro
Source: PLoS One. 2015 Mar 2;10(3):e0115738. doi: 10.1371/journal.pone.0115738 (PMC4346585; doi:10.1371/journal.pone.0115738)
Supplement: S1 Table — (PDF) [file pone.0115738.s002.pdf]

Supplementary Table S1: Oligonucleotides used as Primers in the reported applications. Sequence and position of the primers were from GeneBank Sequence *NG_000006.1

Name	Direction, Sequence	Position from the cap site	Used with primer	Amplicon length (bp)	Application	Refs
A	F-GCCCTGAGCGACCTGCACGCG	α1 & α2 +401/+421	B	α1 301 α2 294	DGGE DNA	[16]
B	R-ACAGAAGCCAGGAACTTGTC	α1 +682/+701; α2 +675/+694				[16]
C	F-GGCAAGAAGGTGGCCGAC	α1 & α2 +332/+349	B	363	DGGE cDNA α2	(Harteveld, *et al* 1996)
D	F-CACAGACTCAGAGAGAACC	α1 & α2 +15/+33	G, H	α1 764 cDNA α1 493	RT-PCR α1, α2	(Foglietta, *et al* 1996)
E	R-GGGGGGAGGCCCAAGGGGCAAGAA	α1 +489/+512	A	cDNA α1 230	RE cDNA analysis	Present article
F	F-TGACCCTCTTCTCTGCACAGCTC	α1 & α2 +584/+606	G	190	RE DNA analysis	[19]
G	R-GAGGCCCAAGGGGCAAGAAGCAT	α1 +751/+773			Seq α1, RT-PCR α1, RE cDNA analysis	[15]
H	R-GGGAGGCCCATCGGGCAGGAGGAAC	α2 +744/+768	A	cDNA α2 226	RT-PCR α2, Acrylamide gel cDNA	[19]
I	F-CACCCCTGCGGTGCACcCCc	α2 +678/+698	J	255	ARMS α2 Hb Policoro	Present article
J	R-GTCTGAGACAGGTAAACACCTCCAT	34642-34618*				[15,17]
K	F-CCCTGCGGTGCACGgCt	α2 +681/+698	L	139	ARMS α2 cod 124 nor	Present article
L	R-CAAGGAGGGGAGGAGGGCCCGTT	α2 +798/+820				Present article
M	F-AGGCTGTGGGCAGAGTCAGAAGA	35943-35965*	N	714	Control ARMS	[15]
N	R-CAATAGCTGGAACCGGCTGGAG	36656-36635*			Control ARMS	[15]
O	F-CCCAGAGCCAGGTTTGTTTATCTG	32840-32863*	J	1803	RFLP RsaI	Present article

F=Forward; R=Reverse. Nucleotides in lower-case letters were the substitutions introduced for ARMS-PCR.

References

Harteveld KL, Heister AJ, Giordano PC, Losekoot M and Bernini LF (1996) Rapid detection of point mutations and polymorphisms of the alpha-globin genes by DGGE and SSCA. Hum Mutat 7: 114-122.

Foglietta E, Deidda G, Graziani B, Modiano G and Bianco I (1996) Detection of alpha-globin gene disorders by a simple PCR methodology. Haematologica 81: 387-396.
